# Conservative versus invasive management of symptomatic hydronephrosis in pregnancy: maternal and fetal outcomes

**DOI:** 10.1186/s12884-026-08824-9

**Published:** 2026-02-14

**Authors:** Nuh Aldemir, İbrahim Üntan, Ipek Uzaldi, Nur Cansu Yılmaz Baldan, Royale Seferli Aldemir

**Affiliations:** 1 Department of Urology, Esenler Hospital, Medipol University, Istanbul, Türkiye; 2 Department of Urology, School of Medicine, Ahi Evran University, Kirsehir, Türkiye; 3 Department of Obstetrics and Gynecology, Esenler Hospital, Medipol University, Istanbul, Türkiye; 4 Department of Obstetrics and Gynecology, Gaziosmanpasa Training and Research Hospital, Istanbul, Türkiye; 5 Kervansaray Mah. 2019. Sok. No:1, Kirsehir, 40100 Türkiye

**Keywords:** Pregnancy, Hydronephrosis, Conservative treatment, Ureteral stents, Birth weight, Pregnancy outcome

## Abstract

**Objective:**

The management of symptomatic hydronephrosis during pregnancy requires balancing maternal and fetal health. This exploratory retrospective study evaluated outcomes of conservative versus invasive intervention approaches in pregnant patients with symptomatic hydronephrosis.

**Methods:**

We conducted a retrospective analysis of pregnant patients with symptomatic hydronephrosis who underwent conservative management or invasive interventions. Groups were compared regarding demographics, gestational age at diagnosis, fetal birth weight, maternal renal function, and pregnancy-related complications.

**Results:**

The conservative (*n* = 52) and invasive intervention (*n* = 29) groups had comparable baseline characteristics, including maternal age (25.8 ± 4.7 vs. 27.0 ± 4.9 years, *p* = 0.290) and gestational age at diagnosis (24.1 ± 5.9 vs. 24.8 ± 5.8 weeks, *p* = 0.610). Birth weight was significantly higher with conservative management (3,289 ± 531 g vs. 3,045 ± 337 g, *p* = 0.029). Multivariable regression analysis adjusting for maternal age, gestational week, and gravidity showed no significant independent association with birth weight (β = 167.5 g, 95% CI: -55.5–390.5 g, *p* = 0.139), with the most significant difference observed in Grade 3 hydronephrosis (654 g, *p* = 0.055). Serum BUN levels were lower with invasive intervention (6.9 ± 1.0 vs. 7.5 ± 1.1 mg/dL, *p* = 0.020), though serum creatinine, a more specific renal function marker, showed no significant difference (*p* = 0.836). Rates of gestational hypertension and preeclampsia were comparable between groups.

**Conclusion:**

Both conservative and invasive management strategies appear feasible and clinically acceptable for symptomatic hydronephrosis during pregnancy, with no clinically significant differences in maternal or neonatal outcomes. Although a statistically significant difference in birth weight was observed, neonatal outcomes, including Apgar scores, were comparable between groups, and birth weights did not fall into categories of small for gestational age or fetal growth restriction. Invasive procedures may be necessary in severe cases with refractory symptoms, infection, or deteriorating renal function. Treatment selection should be individualized based on clinical severity rather than expected outcome differences.

## Brief summary

Both conservative and invasive management of symptomatic hydronephrosis during pregnancy demonstrate comparable maternal and neonatal outcomes. While a statistically significant difference in birth weight was observed, it was not clinically meaningful, as neonatal outcomes were similar between groups. Treatment selection should be guided by clinical severity and individual patient factors.

## Introduction

Symptomatic hydronephrosis (HN) is one of the most common non-obstetric causes of hospitalization during pregnancy and significantly increases the risk of maternal and fetal complications [1]. Unlike physiological HN, which affects over 90% of pregnant women and typically remains asymptomatic, symptomatic HN requires clinical intervention and careful management to ensure optimal maternal and fetal outcomes.

Pregnancy induces anatomical and physiological changes in the urinary tract, particularly in the upper collecting system [2]. Elevated progesterone levels relax ureteral smooth muscle and decrease peristalsis, while mechanical compression from the gravid uterus contributes to urinary stasis [1, 2]. These changes may lead to symptomatic HN or renal colic, which occurs in approximately 1 in 1,500 to 3,500 pregnancies, with symptoms typically manifesting in the second or third trimester [3]. The management of symptomatic HN during pregnancy presents unique challenges due to physiological changes affecting diagnosis and treatment, teratogenic concerns, and the need to balance maternal and fetal safety [4].

Conservative management, including intravenous hydration, analgesics, antispasmodics, and antibiotic therapy when indicated, successfully resolves symptoms in 50% to 60% of cases [5]. However, patients who fail conservative treatment or present with progressive symptoms, refractory pain, infection, or deteriorating renal function may require invasive interventions such as double-J ureteral stent (DJ stent) placement, ureteroscopy (URS), or percutaneous nephrostomy [6].

Despite the availability of both conservative and invasive approaches, there is limited direct evidence comparing maternal and fetal outcomes between these strategies [1, 5]. Most existing studies focus on technical feasibility and immediate complications, with few examining comprehensive obstetric and neonatal outcomes [4]. Understanding the comparative effectiveness of these approaches is essential for optimizing patient selection and improving clinical decision-making.

This retrospective study aimed to evaluate and compare maternal and fetal outcomes associated with conservative versus invasive management of symptomatic HN during pregnancy, with particular focus on fetal birth weight, maternal renal function, and pregnancy-related complications.

## Methods

### Study design and setting

**Fig. 1:**
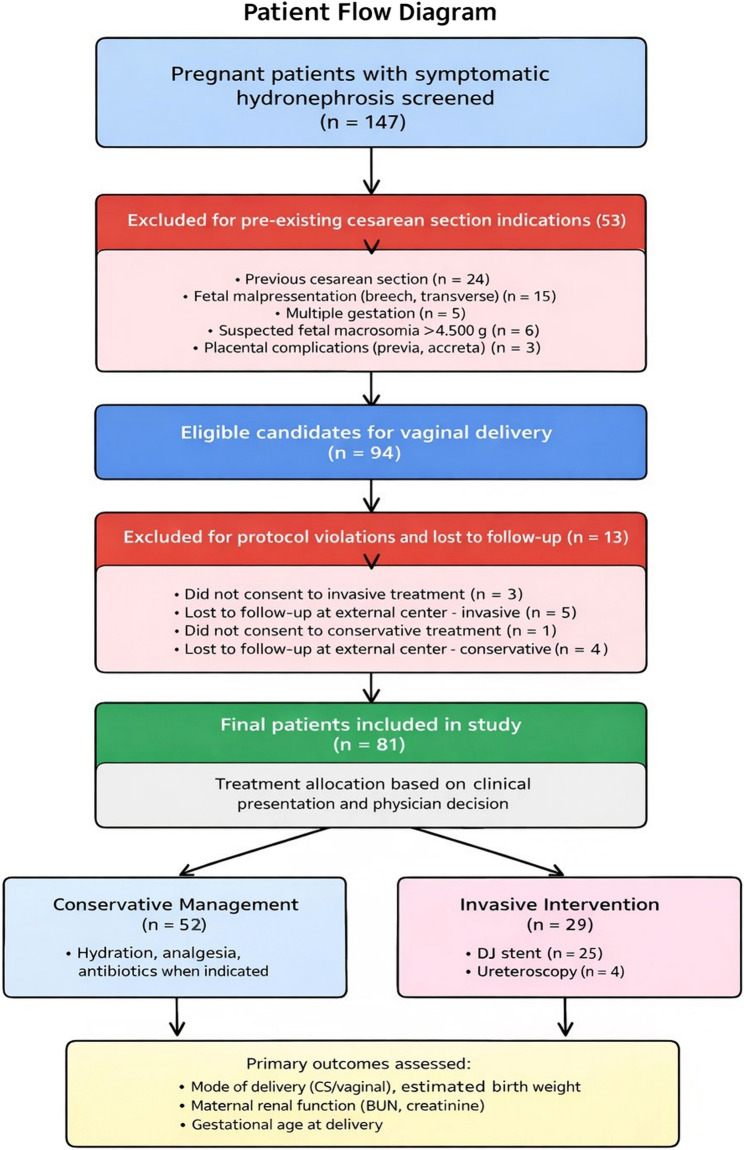
Patient Flow Diagram CONSORT-style flow diagram showing patient selection. Excluding patients with pre-existing cesarean indications ensured that the impact of urological management on mode of delivery could be assessed independently of obstetric factors

This retrospective cohort study screened 29,400 pregnant patients presenting to urology and obstetrics outpatient clinics between October 2017 and December 2024 (Fig. 1). Patients undergoing urological procedures or deliveries at external centers were excluded. Eighty-one patients with symptomatic HN who completed follow-up and delivery at our hospital comprised the study cohort. Treatment allocation was not randomized and reflected real-world clinical decision-making based on symptom severity, response to conservative therapy, and presence of complications.

### Study outcomes and definitions

This exploratory, retrospective study evaluated fetal and maternal outcomes associated with conservative versus invasive management. Primary outcomes were fetal birth weight and maternal renal function markers (blood urea nitrogen [BUN] and serum creatinine). Secondary outcomes included gestational age at delivery, mode of delivery (cesarean section), preterm birth, pregnancy complications (gestational hypertension [GH], preeclampsia [PE], gestational diabetes mellitus [GDM]), urinary tract infections (UTIs), hematuria, and neonatal outcomes (Apgar scores). These outcomes were defined retrospectively based on available clinical data, as this was an exploratory, hypothesis-generating study rather than a confirmatory trial with prospectively registered endpoints.

### Data collection

Patient records were reviewed for age, symptoms, laterality of renal colic, degree and localization of pelvicalyceal ectasia on ultrasonographic evaluation, laboratory findings (white blood cell [WBC] count, BUN, creatinine, urinalysis), gestational age at diagnosis and delivery, birth weight, Apgar scores, delivery mode, GH/PE, and cesarean section indications. GDM was assessed as pregnancy represents a physiological stress state that may unmask glucose intolerance. Pregnancy complications assessed included UTI, hematuria, GH, and GDM.

### Patient selection

Patients with pre-existing indications for planned cesarean delivery were excluded, including previous cesarean section, fetal malpresentation (breech, transverse lie), multiple gestation, suspected macrosomia (estimated fetal weight > 4,500 g), and placental complications (placenta previa, accreta). Only patients eligible for vaginal delivery at enrollment were included, allowing assessment of whether urological management influenced subsequent cesarean delivery need.

### Diagnostic criteria

Renal colic diagnosis was based on clinical presentation (flank pain, nausea, vomiting) and microscopic hematuria on urinalysis. Transabdominal ultrasonography identified HN and assessed severity. Cases without documented pre-pregnancy HN or renal colic were classified as physiological HN of pregnancy and excluded. GH was defined as systolic blood pressure ≥ 140 mmHg and/or diastolic blood pressure ≥ 90 mmHg measured on two occasions at least 4 h apart after 20 weeks of gestation, and PE was diagnosed when GH was accompanied by proteinuria (≥ 300 mg in a 24-hour urine collection or a protein/creatinine ratio ≥ 0.3) or other maternal organ dysfunction [7, 8]. Microscopic hematuria was defined as > 5 red blood cells per high-power field (HPF), and UTI as > 5 WBCs per HPF [1, 9]. HN severity was graded ultrasonographically based on calyceal diameter measurements: Grade 1 (mild) – 5–10 mm; Grade 2 (moderate) – 10–15 mm; Grade 3 (severe) – >15 mm [10]. This grading system is a descriptive classification based on general ultrasonographic criteria and has not been specifically validated for pregnancy-related hydronephrosis.

### Management protocols

Conservative treatment comprised hydration, analgesics, and antibiotic therapy when bacterial infection was present. Urological interventions (DJ stent placement, URS) were reserved for renal colic refractory to pharmacological treatment, persistent symptoms despite adequate conservative management, UTI with obstruction, deteriorating renal function, sepsis, or solitary kidney obstruction. Treatment decisions were individualized by the attending urologist based on clinical presentation, symptom severity, and response to conservative measures. Conservative management was outpatient-based with scheduled follow-up; invasive procedures were performed as day-case or overnight-stay procedures.

### Surgical technique

URS and DJ stent placement were performed under spinal or local anesthesia using a STORZ semi-rigid 7 CH ureteroscope. A 4.8 CH DJ stent (26–28 cm; Plastimed, Tuzla, Turkiye) was retrogradely inserted over a guidewire. Stent positioning was confirmed by direct visualization of stent markers and the distal coil, and by intraoperative ultrasonographic verification within the pyelocaliceal system. Fluoroscopy was not used. Patients were divided into conservative management (*n* = 52) and invasive intervention (*n* = 29) groups based on treatment received.

### Statistical analysis

Data normality was assessed using the Kolmogorov-Smirnov test. Normally distributed variables were reported as mean ± standard deviation; non-normally distributed variables as median with interquartile range. Independent group comparisons used Student’s t-test for parametric data and Mann-Whitney U test for non-parametric data. Categorical variables were analyzed using Pearson’s chi-square or Fisher’s exact test. Multivariable linear regression analysis adjusted for confounding variables, with fetal birth weight as the dependent variable and treatment group, maternal age, gestational week at admission, and gravidity as independent variables. Covariates were selected a priori based on established associations with birth weight and pregnancy outcomes.

## Results

### Baseline characteristics

Baseline characteristics were comparable between groups (Table 1). Maternal age was 25.8 ± 4.7 years in the conservative group and 27.0 ± 4.9 years in the invasive group (*p* = 0.290). Gestational age at presentation was 24.1 ± 5.9 weeks and 24.8 ± 5.8 weeks, respectively (*p* = 0.610). Median gravidity was 1 (1–4) in both groups (*p* = 0.420), and median parity was 0 (0–3) versus 0 (0–2) (*p* = 0.142).

**Table 1:** Clinical and demographic characteristics of the Conservative and invasive intervention groups

Parameter	Conservative (*n* = 52)	Invasive (*n* = 29)	*P* value
Maternal age (years)	25.8 ± 4.7	27.0 ± 4.9	0.290ᵗ
Gestational week at first admission	24.1 ± 5.9	24.8 ± 5.8	0.610ᵗ
Gravidity, median (range)	1(1–4)	1(1–4)	0.420ᶻ
Parity, median (range)	0(0–3)	0(0–2)	0.142ᶻ

Data are presented as mean ± standard deviation (SD) or median (minimum-maximum)

ᵗ Independent sample t-test; ᶻ Mann-Whitney U test

### Obstetric and neonatal outcomes

**Fig. 2:**
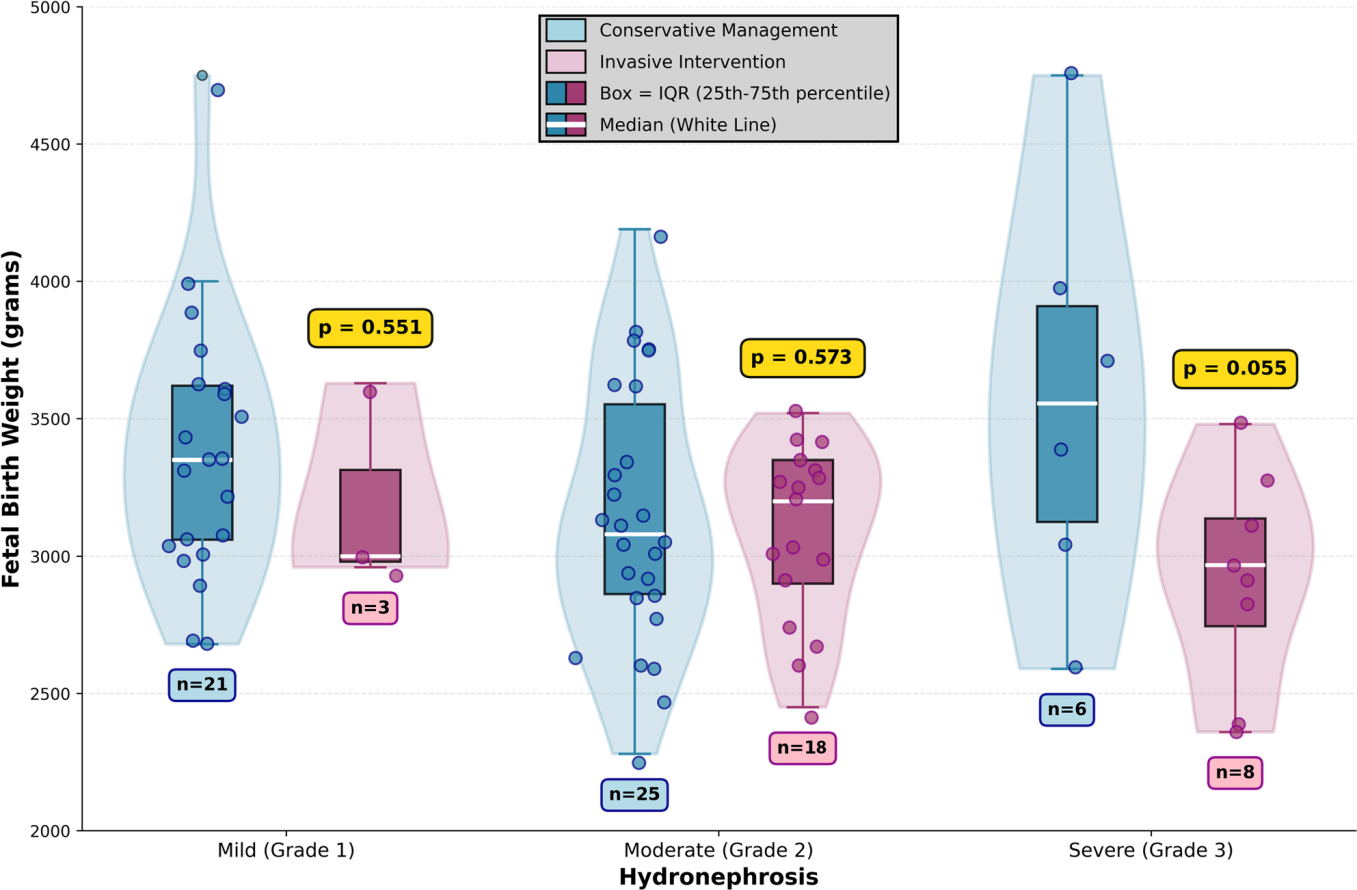
Fetal Birth Weight by Hydronephrosis Grade and Treatment Group.

Gestational age at delivery was 38.2 ± 2.0 weeks in the conservative group and 37.4 ± 1.1 weeks in the invasive group (*p* = 0.072). Preterm birth (< 37 weeks) occurred in 13.5% (7/52) and 17.2% (5/29) of cases, respectively (*p* = 0.747). Median 5-minute Apgar scores were 9 in both groups (*p* = 1.000). Mode of delivery showed no overall difference between groups (*p* = 0.306): vaginal delivery occurred in 31% (16/52) of conservative cases versus 45% (13/29) of invasive cases, while cesarean section occurred in 69% (36/52) versus 55% (16/29), respectively (Table 2). However, among nulliparous patients (*n* = 49), cesarean section rates differed significantly: 76.7% (23/30) in the conservative group versus 36.8% (7/19) in the invasive group (*p* = 0.008). Fetal birth weight was significantly higher in the conservative group: 3,289 ± 531 g versus 3,045 ± 337 g (*p* = 0.029) (Fig. 2). Multivariable linear regression controlling for maternal age, gestational week at admission, and gravidity showed that conservative management was not independently associated with higher birth weight (β = 167.5 g, 95% CI: -55.5–390.5 g, *p* = 0.139) (Table 3). Neither maternal age (β = 1.6 g, *p* = 0.901), gestational week at admission (β = 8.1 g, *p* = 0.390), nor gravidity (β = -32.1 g, *p* = 0.614) showed significant associations with birth weight. Subgroup analysis by HN grade revealed birth weight differences of 177 g in Grade 1 (*p* = 0.551), 75 g in Grade 2 (*p* = 0.573), and 654 g in Grade 3 (*p* = 0.055) (Table 4). Gestational age at delivery did not differ significantly across any grade subgroup (all *p* > 0.05).

**Table 2:** Pregnancy characteristics of the groups

Parameters	Conservative (*n* = 52)	Invasive (*n* = 29)	*P* value
Gestational age at delivery (weeks)	38.2 ± 2.0	37.4 ± 1.1	0.072ᵗ
5-min Apgar score	9	9	1.000ᶻ
Mode of delivery, %			0.306ˣ²
Vaginal	16 (31%)	13(45%)	
Cesarean Section	36 (69%)	16 (55%)	
Fetal birth weight (grams)	3,289 ± 531	3,045 ± 337	0.029ᵗ
GDM, %	3 (5.8%)	2 (6.9%)	1.000ᶠ
GH or PE, %	4 (7.7%)	2 (6.9%)	1.000ᶠ
Preterm birth (< 37 weeks), n (%)	7 (13.5%)	5 (17.2%)	0.747ᶠ

Data are presented as mean ± standard deviation (SD) or count (percentage)

*GDM* gestational diabetes mellitus, *GH* gestational hypertension, *PE* preeclampsia

ᵗ Independent sample t-test; ᶻ Mann-Whitney U test; ˣ² Chi-square test; ᶠ Fisher's exact test

**Table 3:** Multivariable analysis of fetal birth weight multiple linear regression results (*n* = 81)

Variable	β (95% CI)	*p*-value
Treatment group*	167.5 (-55.5, 390.5)	0.139
Maternal age (years)	1.6 (-23.5, 26.7)	0.901
Gestational week at admission	8.1 (-10.5, 26.7)	0.390
Gravidity	-32.1 (-158.1, 93.9)	0.614

*Conservative management compared to invasive intervention (reference group)

Model statistics: R² = 0.065, Adjusted R² = 0.016, Overall *p* = 0.268

### Pregnancy complications

The incidence of gestational diabetes was 5.8% (3/52) in the conservative group and 6.9% (2/29) in the invasive group (*p* = 1.000). GH or PE occurred in 7.7% (4/52) versus 6.9% (2/29) of cases (*p* = 1.000).

### Renal function and laboratory parameters

**Fig. 3:**
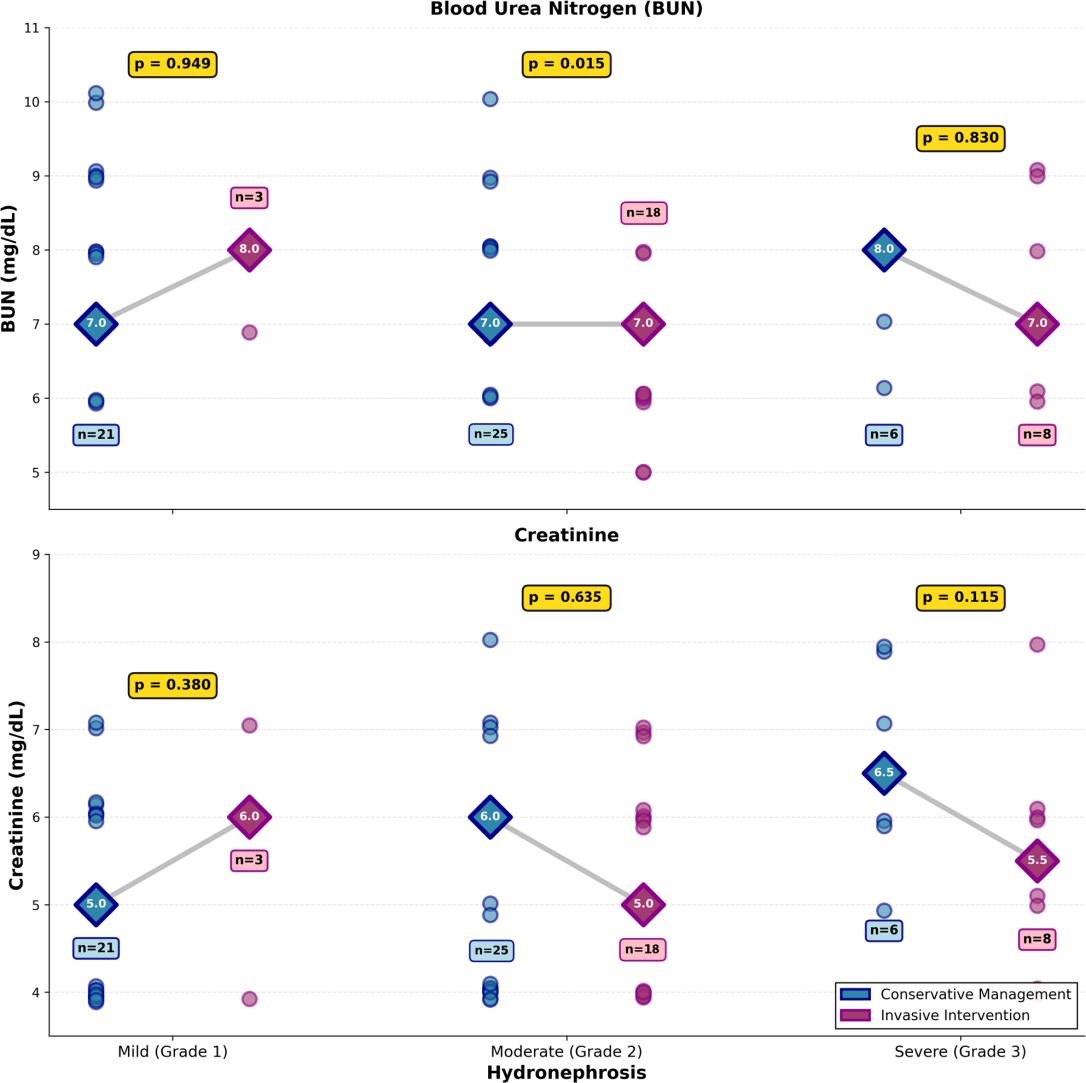
Renal Function Parameters by Hydronephrosis Severity and Treatment Approach. Each data point represents an individual patient. Diamond markers indicate median values. Gray connecting lines illustrate median changes between treatment groups. P-values from Mann-Whitney U tests are displayed in gold boxes

Serum BUN was 7.5 ± 1.1 mg/dL in the conservative group and 6.9 ± 1.0 mg/dL in the invasive group (*p* = 0.020) (Fig. 3). Serum creatinine was 0.5 ± 0.1 mg/dL in both groups (*p* = 0.836). WBCs were 10.2 ± 2.0 × 10³/µL versus 10.0 ± 1.9 × 10³/µL (*p* = 0.618). Subgroup analysis (Table 4) showed BUN levels were significantly lower in the invasive group among Grade 2 patients: 6.6 ± 0.8 versus 7.3 ± 0.9 mg/dL (*p* = 0.015 by independent t-test; *p* = 0.023 by Mann-Whitney U test).

**Table 4:** Subgroup analysis by hydronephrosis grade

Parameter	Grade 1 (*n* = 24)	Grade 2 (*n* = 43)	Grade 3 (*n* = 14)
Sample size			
Conservative	21	25	6
Invasive	3	18	8
Fetal Birth Weight (g)			
Conservative	3,373 ± 481	3,142 ± 482	3,577 ± 756
Invasive	3,196 ± 375	3,081 ± 320	2,923 ± 387
*p*-value	0.551ᵗ	0.573ᵗ	0.055ᵗ
Gestational Age at Delivery (weeks)			
Conservative	38.8 ± 2.7	37.7 ± 1.2	38.0 ± 1.3
Invasive	37.3 ± 0.6	37.7 ± 1.0	37.0 ± 1.3
*p*-value	0.224ᶻ	0.949ᶻ	0.180ᶻ
BUN (mg/dL)			
Conservative	7.7 ± 1.2	7.3 ± 0.9	7.5 ± 0.8
Invasive	7.7 ± 0.6	6.6 ± 0.9	7.4 ± 1.2
*p-*value	0.949ᵗ / 0.928ᶻ	00.015ᵗ / 0.023ᶻ	0.830ᵗ / 0.788ᶻ

Data presented as mean ± SD. ᵗ Independent sample t-test; ᶻ Mann-Whitney U test*. BUN* blood urea nitrogen

### Hydronephrosis characteristics

HN grade distribution differed between groups (*p* = 0.010) (Table 5). Grade 1 was more common in the conservative group (40% vs. 10%), while Grade 3 was more frequent in the invasive group (28% vs. 12%). HN laterality did not differ between groups (*p* = 0.468).

**Table 5:** Clinical and laboratory data of the participants

Parameter	Conservative (*n* = 52)	Invasive (*n* = 29)	*P* value
Serum BUN (mg/dl)	7.5 ± 1.1	6.9 ± 1.0	0.020ᵗ
Serum creatinine (mg/dl)	0.5 ± 0.1	0.5 ± 0.1	0.836ᵗ
WBC (×10^3^/µl)	10.2 ± 2.0	10.0 ± 1.9	0.618ᵗ
HN grade, (%)			0.010ˣ²
Grade 1	21 (40)	3 (11)	
Grade 2	25 (48)	18 (62)	
Grade 3	6 (11)	8 (28)	
HN site, (%)			0.468ˣ²
Right	37	19	
Left	12	6	
Bilateral	4	3	
UTI, (%)	27 (50)	16 (57)	0.961ˣ²
Hematuria (gross/microscopic), (%)	10 (19)	8 (29)	0.556ˣ²

Data are presented as mean ± standard deviation (SD) or count (percentage)

*BUN* blood urea nitrogen, *WBC* white blood cell, *HN* hydronephrosis, *UTI* urinary tract infection

Statistical significance was set at *p* < 0.05

ᵗ Independent sample t-test; ˣ² Chi-square test

### Urological complications

UTI incidence was 52% (27/52) in the conservative group and 55% (16/29) in the invasive group (*p* = 0.961). Hematuria (gross or microscopic) occurred in 19% (10/52) versus 28% (8/29) of cases (*p* = 0.556).

### Intervention characteristics (Invasive Group)

Among 29 patients receiving invasive intervention, 25 (86%) underwent DJ stent placement, and 4 (14%) underwent URS (Table 6). Stent placement was performed in 72% (18/25) of right-sided, 16% (4/25) of left-sided, and 12% (3/25) of bilateral interventions. URS was performed in 25% (1/4) of right-sided and 75% (3/4) of left-sided interventions. The association between laterality and intervention type was statistically significant (*p* = 0.037). Intervention type distribution by HN severity showed no significant association (*p* = 0.735) (Table 7). Stent placement was performed in 12.0% (3/25) of Grade 1, 60.0% (15/25) of Grade 2, and 28.0% (7/25) of Grade 3 cases. URS was performed in 0% of Grade 1, 75% (3/4) of Grade 2, and 25% (1/4) of Grade 3 cases.

**Table 6:** Comparison of intervention techniques and laterality

Laterality	DJ Stent, *n* (%)	URS, *n* (%)
Right	18 (72%)	1 (25%)
Left	4 (17%)	3 (75%)
Bilateral	3 (12%)	0 (0%)
Total	25 (100%)	4 (100%)

Analysis performed using Fisher’s exact test (*p* = 0.037). The small number of URS cases (*n* = 4) limits statistical power and interpretability of comparisons between intervention types. *URS* Ureteroscopy

**Table 7:** Intervention type by hydronephrosis severity

Hydronephrosis Grade	DJ Stent (*n* = 25) n (%)	URS (*n* = 4) n (%)	Total (*n* = 29) n (%)	*p*-value
Grade 1	3 (12.0)	0 (0.0)	3 (10.3)	0.735
Grade 2	15 (60.0)	3 (75.0)	18 (62.1)	
Grade 3	7 (28.0)	1 (25.0)	8 (27.6)	

Data presented as n (%). The chi-square test was used to compare the distribution of intervention types across hydronephrosis grades. The small number of URS cases (*n* = 4) limits statistical power and interpretability of this comparison

## Discussion

Baseline characteristics were comparable between treatment groups, with no differences in maternal age, gestational age at presentation, gravidity, or parity. This comparability strengthens the validity of outcome comparisons, as clinical factors such as symptom severity and complications, rather than demographic characteristics, determined treatment selection [11, 12].

Although a statistically significant difference in birth weight was observed between groups, this finding must be interpreted with caution, given the inherent selection bias in this retrospective study. The invasive management group represented a clinically more severe subgroup with limited or no alternative treatment options. Consequently, any comparison of outcomes must account for the fact that treatment allocation was determined by disease severity rather than randomization. Importantly, the birth weight difference, while statistically significant, did not translate into clinically meaningful differences in neonatal outcomes: Apgar scores were identical between groups, and neonatal weights did not fall into categories of small for gestational age or fetal growth restriction. These findings support the interpretation that neonatal outcomes are similar between management strategies. The observed birth weight difference likely reflects indication and severity bias rather than a direct treatment effect, and the results should not be extrapolated to suggest that invasive management itself is associated with worse neonatal outcomes [13, 14]. Previous studies have reported reassuring fetal outcomes following invasive interventions performed during pregnancy [15, 16]. This may reflect successful patient selection, with more severe cases appropriately receiving invasive intervention.

Subgroup analysis by HN grade showed the largest birth weight differences in severe cases. Although the difference in Grade 3 HN did not reach statistical significance, this may reflect the limited sample size in this subgroup. The observed magnitude of the difference is noteworthy but should be interpreted cautiously given the exploratory nature and small sample size of this subgroup analysis. This exploratory finding warrants further investigation in larger prospective studies and may inform future research on treatment selection in high-grade obstruction [11, 17]. The comparable outcomes in Grade 2 cases suggest both treatment approaches may yield similar outcomes in moderate HN, allowing clinicians to base management decisions on individual patient factors.

Apgar scores were identical between groups, indicating both treatment methods had similarly positive effects on fetal health. Studies have observed high Apgar scores in both conservative and surgical treatment groups, supporting that ureteral stenting does not adversely affect fetal mortality or morbidity [16, 18]. Subgroup analysis of nulliparous patients revealed a statistically significant difference in cesarean section rate in the invasive intervention group, raising the hypothesis that effective relief of urinary obstruction might be associated with reduced need for cesarean delivery in this population; however, this exploratory finding should be interpreted with caution given the small subgroup sample size. This unexpected finding merits further investigation, as the mechanism by which urological intervention might influence the mode of delivery remains unclear.

Gestational diabetes and PE rates were similar between groups, indicating both treatment approaches have comparable effects on pregnancy complications. Although gestational age at delivery did not differ significantly, the trend toward earlier delivery in the invasive group may reflect underlying disease severity requiring intervention. More severe maternal conditions have been associated with increased preterm birth risk [4, 5]. Our data suggest that disease severity, rather than treatment type, is the primary determinant of delivery timing.

Although the invasive intervention group had statistically lower BUN levels than the conservative management group, BUN should not be interpreted as a reliable renal outcome measure during pregnancy. BUN levels are significantly influenced by hydration status, dietary protein intake, and the physiological changes of pregnancy, making them an unreliable indicator of renal function in this population [5, 9]. Notably, serum creatinine, a more specific marker of renal function, did not differ between groups. Overall, the renal outcomes do not demonstrate a meaningful difference between conservative and invasive management. Relief of urinary obstruction through invasive intervention may reduce kidney burden, but this potential benefit must be weighed against the invasive nature and potential complications.

The distribution of HN severity differed significantly between treatment groups, with the conservative group having milder cases and the invasive group having more severe cases. This pattern reflects clinical practice, in which invasive intervention is reserved for more severe HN that requires intensive management. These findings indicate disease severity, rather than random allocation, drove treatment selection in this retrospective cohort. Stent placement is typically preferred in severe HN cases that are not responding to conservative treatment. However, complications of stent placement, such as infection and the risk of stone formation, should be considered [16, 18].

UTI incidence was comparable between groups, indicating that the treatment approach does not significantly affect infection risk. UTIs are common in pregnant women with urolithiasis regardless of management strategy, posing potential threats to maternal and fetal health [18]. The high overall UTI incidence underscores the importance of vigilant monitoring and prompt treatment. While invasive interventions theoretically could increase infection risk through instrumentation, our data do not demonstrate this effect, possibly reflecting appropriate antibiotic prophylaxis and careful technique. Hematuria incidence showed a non-significant trend toward higher rates following invasive intervention, as expected, given that hematuria is a common side effect of invasive urological procedures [19].

Regarding laterality patterns, ureteral stent placement was more common on the right side, while URS was more frequently performed on the left, consistent with the literature [16]. Ultrasound-guided URS has been reported as a safe option during pregnancy [16, 20, 21]. The higher proportion of patients undergoing URS on the left side supports the feasibility of this technique in selected cases. However, the limited number of URS procedures limits the interpretability of these findings, and more clinical data are needed on patient selection and maternal-fetal safety.

This study has several limitations. As a retrospective, non-randomized study, treatment allocation was based on clinical judgment rather than random assignment, introducing potential selection bias and confounding by indication. Patients with more severe symptoms were more likely to receive invasive intervention, while those with milder presentations were managed conservatively. Although multivariable regression analysis adjusted for measured confounders, unmeasured factors may still influence observed associations between treatment approach and outcomes. The higher birth weight in the conservative group may reflect underlying differences in disease severity and patient characteristics rather than direct treatment effect. Causal inferences should be made with caution, and findings should be interpreted as associations rather than causal relationships. The sample size represents the available cohort during the study period, limiting the ability to detect small effect sizes for some secondary outcomes. Multiple statistical comparisons were performed without adjustment for multiple testing, potentially increasing the Type I error rate. However, this approach is standard for exploratory retrospective studies aimed at hypothesis generation. Our findings are biologically plausible and consistent with existing literature. Maternal baseline body weight and gestational weight gain were not systematically recorded, limiting assessment of their influence on fetal birth weight. Detailed urinary stone characteristics were not systematically documented, restricting analysis of relationships between stone etiology and intervention choice. Outcomes were not prospectively defined before data collection, limiting the confirmatory nature of findings. This study should be considered exploratory and hypothesis-generating, and its findings should be validated in prospective studies with predefined endpoints. The lack of long-term follow-up data limits the evaluation of the long-term effects of treatment on fetal and maternal health. The small number of URS procedures limits subgroup analyses comparing intervention types. The single-center design limits generalizability, and multicenter studies would better assess the impact of different clinical practices.

## Conclusions

This exploratory retrospective study found no clinically significant differences in maternal or neonatal outcomes between conservative and invasive management of symptomatic HN during pregnancy. Both approaches appear feasible and safe, with treatment selection appropriately guided by clinical severity and individual patient factors. While a statistically significant difference in birth weight was observed, neonatal outcomes, including Apgar scores, were similar between groups, and no clinically relevant differences in neonatal morbidity were identified. Renal function, as measured by serum creatinine, did not differ between groups; BUN differences should not be interpreted as a meaningful renal outcome given the limitations of this marker in pregnancy. The invasive management group represented patients with more severe disease who required intervention due to refractory symptoms, infection, or deteriorating renal function; the observed outcomes in this group should not be interpreted as adverse effects of invasive treatment itself. Treatment decisions should be individualized based on the patient’s clinical condition, gestational age, symptom severity, and the balance between maternal and fetal considerations. Future large-scale, multicenter, prospective studies could provide more definitive evidence for the management of urolithiasis in pregnancy, ultimately improving patient care.

## Data Availability

The datasets supporting the current study’s findings are openly available at https://doi.org/10.5281/zenodo.18437815.
